# Vogt‐Koyanagi‐Harada disease‐like posterior uveitis in the course of nivolumab (anti‐PD‐1 antibody), interposed by vemurafenib (BRAF inhibitor), for metastatic cutaneous malignant melanoma

**DOI:** 10.1002/ccr3.911

**Published:** 2017-03-31

**Authors:** Toshihiko Matsuo, Osamu Yamasaki

**Affiliations:** ^1^ Department of OphthalmologyOkayama University Hospital and Graduate School of Medicine, Dentistry, and Pharmaceutical SciencesOkayama CityJapan; ^2^ Department of DermatologyOkayama University Hospital and Graduate School of Medicine, Dentistry, and Pharmaceutical SciencesOkayama CityJapan

**Keywords:** BRAF inhibitor, nivolumab, PD‐1, uveitis, vemurafenib, Vogt‐Koyanagi‐Harada disease

## Abstract

A patient with metastatic cutaneous malignant melanoma developed Vogt‐Koyanagi‐Harada disease‐like posterior uveitis after two nivolumab (anti‐PD‐1 antibody) injections. Vogt‐Koyanagi‐Harada disease, with the background of autoimmunity against choroidal melanocytes, suggests nivolumab be working by disintegrating inhibition circuit of T cells against a common epitope shared between melanoma cells and normal melanocytes.

## Introduction

Vogt‐Koyanagi‐Harada disease is a bilateral, diffuse granulomatous uveitis associated with poliosis, vitiligo, alopecia, and central nervous system and auditory signs 1. Autoimmune mechanisms, directed against melanocytes, are considered to underlay the inflammation 1, 2.

Nivolumab, a human immunoglobulin G4 (IgG4) monoclonal antibody against human programmed death receptor‐1 (PD‐1), has been recently introduced as a targeted therapy for unresectable or metastatic melanoma 3. In this report, we present a patient with metastatic malignant melanoma who developed Vogt‐Koyanagi‐Harada disease‐like posterior uveitis in the course of nivolumab administration.

## Case Report

**Figure 1:**
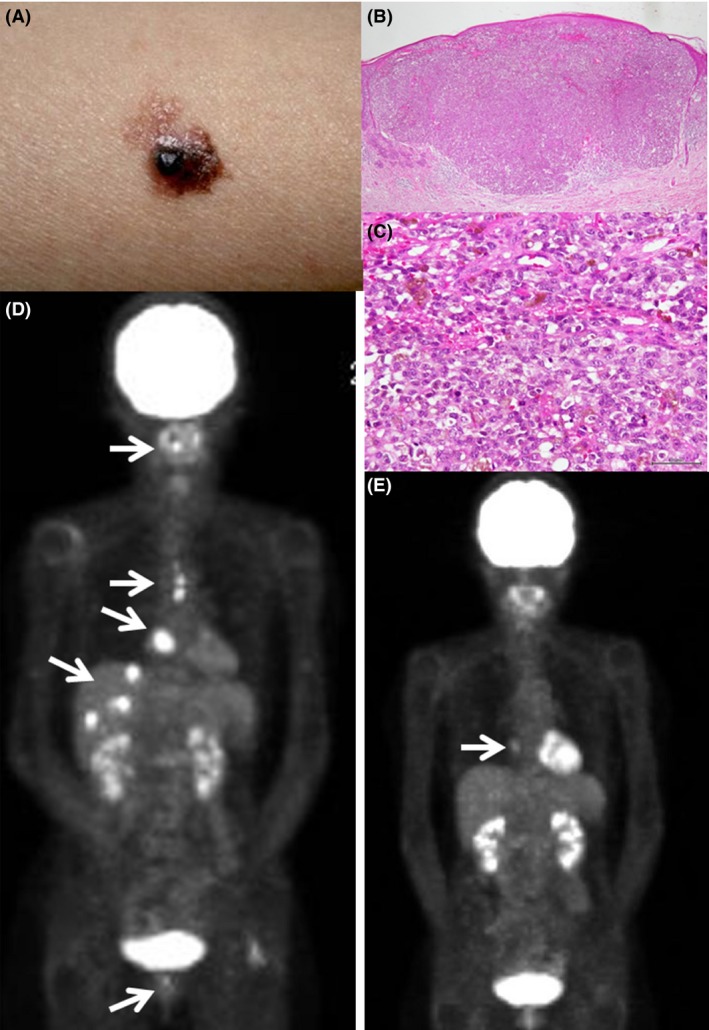
Blackish skin lesion with irregularly surfaced elevation (A) on flexor part of the right forearm of a 60‐year‐old woman in April 2013, which is proven histopathologically as malignant melanoma (x4 low magnification in B with bar = 500 *μ*m, and x40 high magnification in C with bar = 50 *μ*m). Multiple metastatic lesions (D) in the liver with maximum standardized uptake values (SUVmax), ranging from 7.36 to 8.58, together with bony metastases in the sternum (SUVmax = 5.70), cervical (C2) vertebrate (SUVmax = 6.64), and right pubic bone (SUVmax = 3.83), also with a high‐uptake site at the right atrium of the heart (SUVmax = 7.66) in whole‐body 2‐[^18^F]fluoro‐2‐deoxy‐D‐glucose (FDG) positron emission tomography fused with computed tomography (PET/CT) on March 10, 2015 (arrows in D). PET/CT on September 29 shows only an atrium lesion (arrow in E) with reduced uptake (SUVmax = 2.94), but no hepatic or bony lesions (E).

A 60‐year‐old woman had 10‐year history of a light brownish spot, 1–2 cm in size, on the flexor part of the right forearm. She noticed blackish irregularly surfaced elevation of the skin lesion (Fig. 1A) in April 2013 and visited a hospital. With histopathological diagnosis of malignant melanoma by biopsy (Fig. 1B and C), she underwent the total resection of the lesion, together with ample surrounding normal skin margin, in May.

In July, she began to have local skin injection of natural‐form interferon‐β once in a week initially, and later once in a month. She was well with no abnormalities on computed tomographic scans, covering the neck, thorax, abdomen to the pelvis, once in half a year until the end of September 2014, when routine blood tests showed the rise of serum 5‐S‐cysteinyldopa to 33.8 nmol/L. In the end of November, two high‐uptake sites at axillary lymph nodes on the right side were disclosed by whole‐body 2‐[^18^F]fluoro‐2‐deoxy‐D‐glucose (FDG) positron emission tomography fused with computed tomography (PET/CT). She underwent axillary lymph nodes dissection on the right side, with pathological diagnosis of metastatic melanoma. Local interferon injection was no more given afterward.

In the beginning of March 2015, she underwent cataract surgery with intraocular lens implantation on the right eye at a local eye doctor. On March 10, PET/CT detected multiple metastatic lesions in the liver with maximum standardized uptake values (SUVmax), ranging from 7.36 to 8.58, together with bony metastases in the sternum (SUVmax = 5.70), cervical (C2) vertebrate (SUVmax = 6.64), and right pubic bone (SUVmax = 3.83), also with a high‐uptake site at the right atrium of the heart (SUVmax = 7.66, Fig. 1D). Head magnetic resonance imaging with contrast enhancement disclosed no metastatic brain lesions. She received intravenous nivolumab (Opdivo) at the dose of 94 mg/body (2 mg/kg body weight) on March 17 and April 7.

She then had oral vemurafenib (Zelboraf, BRAF inhibitor), 1920 mg daily, on April 21, since she was shown at this time to have the BRAF V600E mutation by an FDA‐approved test, The cobas 4800 BRAF V600 Mutation Test (Roche Molecular Diagnostics). Two days later, she developed headache, and 3 days later, she had general arthralgia and systemic skin rashes, like erythema multiforme.

**Figure 2:**
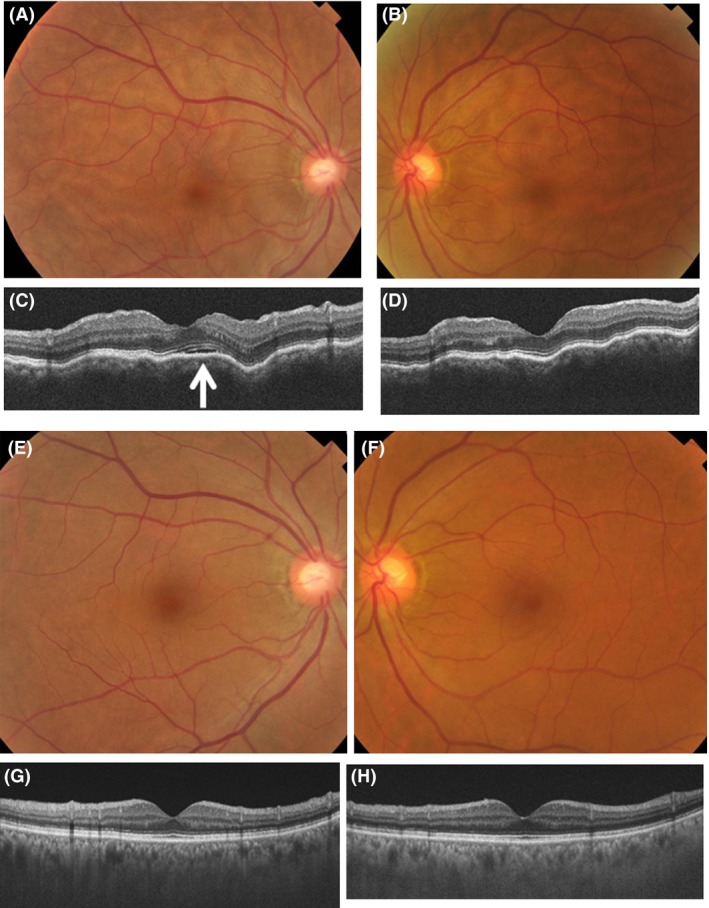
Fundus photographs and vertical sections of optical coherence tomographic scans (right eye in left column and left eye in right column) on April 28, 2015 (top four panels) and May 12 (bottom four panels). Note multifocal bumpy appearance in the posterior pole of the fundus (A and B) and wavy retinal pigment epithelium (C and D) in both eyes, with subretinal fluid (arrow in C) in the right eye on April 28. The fundi in both eyes appear normal (E and F) and optical coherence tomography shows normal lining of the retinal pigment epithelium in both eyes (G and H) on May 12. The inferior side of vertical sections of optical coherence tomography is depicted on the left side of figures.

On April 28, she was referred to an ophthalmologist for xanthopsia (yellow‐colored vision) in both eyes. The best‐corrected visual acuity was 1.5 in both eyes, and the intraocular pressure was 14 mmHg in both eyes. The right eye had intraocular lens implantation while the left eye had no cataract. Both eyes showed no aqueous cells or keratic precipitates. The posterior pole of the retina had multiple bumpy elevations in both eyes (Fig. 2A and B). Optical coherence tomography showed wavy retinal pigment epithelial line with multifocal choroidal thickening in both eyes (Fig. 2C and D), together with subretinal fluid only in the right eye (arrow in Fig. 2C). She had been using 0.1% fluorometholone eye drops four times daily only in the right eye after the cataract surgery. With clinical diagnosis of choroiditis (uveitis) in both eyes at the early stage of Vogt‐Koyanagi‐Harada disease, fluorometholone instillation in the right eye was discontinued and was replaced with 0.1% betamethasone eye drops four times daily in both eyes.

Oral vemurafenib was discontinued on April 30, and oral prednisolone was tapered from 30 mg daily on May 1 and discontinued on May 13. The retinal bumpy elevations disappeared in both eyes (Fig. 2E and F), and the normal lining of the retinal pigment epithelium was visualized by optical coherence tomography in both eyes (Fig. 2G and H). On May 19, readministration of vemurafenib 1920 mg daily, combined with oral prednisolone 20 mg daily at this time, resulted again in systemic skin rashes. Vemurafenib was discontinued next day, and prednisolone 30 mg daily was tapered. The skin rashes resolved and she continued to have no abnormalities in both eyes. On May 22, computed tomographic scan demonstrated the reduction of metastatic lesions in the liver and bones.

**Figure 3:**
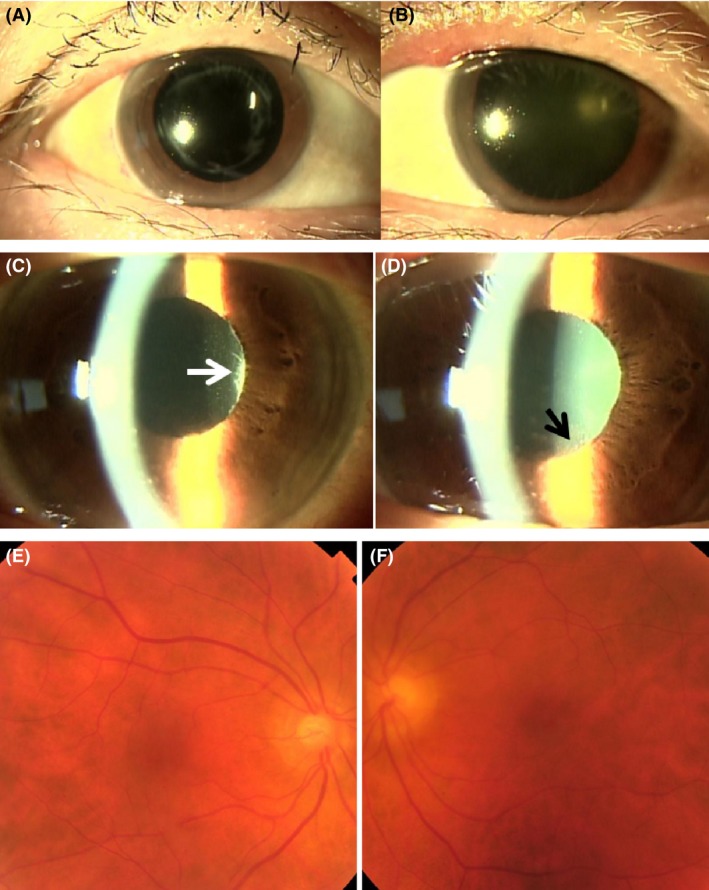
White depigmented eyelashes in the right eye (A) and left eye (B), with mascara, in July 2015. Intraocular inflammation in the anterior segment of the right eye (C) and left eye (D), presenting as mutton‐fat keratic precipitates and white iris nodules along the pupillary margin (arrows) in April 2016, two months after 0.1% betamethasone eye drops have been discontinued due to steroid‐induced intraocular pressure elevation. The right eye only has intraocular lens implantation. She also shows red depigmented fundus in the right eye (E) and left eye (F).

On June 16, she received third injection of nivolumab 94 mg/body. Around that time, she noticed that eyelashes and eyebrows on both sides became white (Fig. 3A and B), as late sequelae in Vogt‐Koyanagi‐Harada disease. She continued to use 0.1% betamethasone eye drops in both eyes. Every 3 weeks, she continued to receive sequential injections of nivolumab 94 mg/body. She experienced no recurrence of intraocular inflammation in both eyes on these occasions. On September 29, PET/CT (Fig. 1E) disclosed that the metastatic lesions in the liver and the bone disappeared and that the lesion at the right atrium reduced in activity (SUVmax = 2.94). She had no systemic complications and continued every‐3‐week nivolumab injection. She also continued to use 0.1% betamethasone eye drops since temporary substitution of betamethasone with nonsteroidal bromfenac eye drops resulted in anterior chamber inflammation in both eyes, presenting as aqueous cells and keratic precipitates.

In February 2016, she showed the elevation of intraocular pressure in both eyes to 25 mmHg. She did not have intraocular inflammation. With the diagnosis of steroid‐induced intraocular pressure elevation, known as a steroid responder, 0.1% betamethasone eye drops was replaced with nonsteroidal bromfenac eye drops. In March, she showed no intraocular inflammation. Cervix to pelvis computed tomographic scan disclosed neither recurrence nor metastasis of malignant melanoma. In April, she complained of blurred vision in both eyes. She had 2+ mutton‐fat keratic precipitates and whitish iris nodules along the pupillary margin in both eyes (Fig. 3C and D), characteristic of Vogt‐Koyanagi‐Harada disease. At this time, she showed apparent red depigmented fundi of both eyes (Fig. 3E and F), a hallmark of Vogt‐Koyanagi‐Harada disease in the late phase. She resumed 0.1% betamethasone eye drops, leading to the subsidence of intraocular inflammation. She did not develop neurological or auditory symptoms such as headache, hearing disturbance and tinnitus throughout the course. She maintained complete remission, only with monthly nivolumab until the latest visit in February 2017.

## Discussion

Nivolumab binds to PD‐1 on the surface of T cells, and blocks the interaction between PD‐1 and its ligands, PD‐L1, and PD‐L2. PD‐1 ligands are upregulated in some malignancies, and suppressor T cells are activated by PD‐1 ligands which bind to PD‐1 on the T‐cell surface. The activated suppressor T cells, in turn, inhibit the immune surveillance of neoplastic cells, resulting in growth of tumors. By blocking the interaction between PD‐1 and its ligands, T cells are released from an inhibition pathway of the immune surveillance, and play a role again in the immune surveillance of neoplastic cells 3.

In the present patient, malignant melanoma cells and normal choroidal melanocytes would share a target epitope for T‐cell recognition. The release of a T‐cell line from a PD‐1 inhibition pathway would lead T cells to be directed against melanoma cells. The activated T cells would be also directed against normal choroidal melanocytes in this patient.

The multifocal choroidal thickening, observed in this patient, would be caused histopathologically by the accumulation of lymphocytes in the choroid 1, 2. In Vogt‐Koyanagi‐Harada disease, lymphocytes accumulate in the uvea, including the choroid, ciliary body, and iris. In the process of disease progression, the retinal pigment epithelium barrier breaks down, and fluid is accumulated under the sensory retina as subretinal fluid, associated also with intraretinal fluid accumulation 4, 5. A wavy line of retinal pigment epithelium, which is caused by multifocal choroidal thickening lesions, is visualized by optical coherence tomography as an earliest manifestation in Vogt‐Koyanagi‐Harada disease 6. The diagnosis of Vogt‐Koyanagi‐Harada disease in the present patient was further supported by white depigmented eyelashes and eyebrows, and also by red depigmented fundi of both eyes, all of which are hallmarks of the disease at the late stage 1, 2.

Other types of uveitis, including sarcoidosis, posterior scleritis, and uveal effusion, were excluded on the basis of clinical presentations. Based on the diagnostic criteria of the disease 7, the present patient would be more accurately diagnosed as Vogt‐Koyanagi‐Harada disease‐like posterior uveitis as the patient was not confirmed to have neurological and auditory signs. The diagnostic criteria also require no history of penetrating ocular trauma before the onset of uveitis. Preceding cataract surgery only in the right eye of the patient was not usually taken as history of penetrating ocular trauma in the background of bilateral uveitis.

Uveitis, although low (presumably less than 1%) in the incidence, is on the list of adverse events for nivolumab (Opdivo prescribing information of the package insert). To the best of our knowledge, this case is the first to show that nivolumab would lead to the development of Vogt‐Koyanagi‐Harada disease‐like posterior uveitis, as a more specific type in the broad entity of uveitis. As Vogt‐Koyanagi‐Harada disease is considered as an autoimmune disease, directed against melanocytes 1, 2, the development of Vogt‐Koyanagi‐Harada disease‐like posterior uveitis in this patient suggests that nivolumab might be working well to make lymphocytes attack on melanoma cells, together with their attack on normal choroidal melanocytes.

The course in this patient was complicated by vemurafenib administration after the two intravenous injections with nivolumab. The patient developed an allergic reaction to vemurafenib in a few days, presenting as erythema multiforme‐like systemic skin rashes. Oral prednisolone, tapered from 30 mg daily, reduced the allergic manifestations and also resolved the fundus presentations of uveitis in both eyes. Vemurafenib is known to cause uveitis in 4% of patients in clinical trials 8. A mean duration until the onset of uveitis after the start of vemurafenib was 5.6 months, with the range from 19 days to 7 months 8. Based on the previous experiences with vemurafenib 8, 9, 10, 11, 12, uveitis in the present patient, as an adverse event, had too earlier an onset after the administration. In addition, local administration of interferon could not be related with the onset of uveitis as the interferon injection was discontinued after lymph nodes metastases were found.

Vogt‐Koyanagi‐Harada disease‐like posterior uveitis in the present patient would be, thus, attributed mainly to nivolumab administration. The patient, indeed, experienced a few episodes of relapse of bilateral uveitis in the long course only with nivolumab. In another way of interpretation, the preceding administration of nivolumab might, at first, break the inhibition circuit of an immunological reaction against vemurafenib, which might, in turn, lead to an earlier onset of general skin rashes. In Japan, nivolumab has been covered by health insurance reimbursement since September 2014 while vemurafenib has been covered by the reimbursement later in February 2015. Based on this flow of the drug approval and insurance coverage, nivolumab was administered first in the present patient with metastatic malignant melanoma. The sequential administration of nivolumab and vemurafenib might lead to the earlier development of Vogt‐Koyanagi‐Harada disease‐like posterior uveitis in this patient.

Vogt‐Koyanagi‐Harada disease‐like posterior uveitis in this patient subsided in 2 weeks with oral administration of prednisolone for skin rashes which was caused by vemurafenib. The patient experienced no recurrence of uveitis as far as she was using 0.1% betamethasone eye drops in both eyes. When 0.1% betamethasone eye drops were discontinued due to steroid‐induced intraocular pressure elevation, intraocular inflammation in the anterior segment of the eye relapsed as numerous mutton‐fat keratic precipitates with iris nodules, characteristic of Vogt‐Koyanagi‐Harada disease. She also developed depigmentation of eyelashes and eyebrows in the relatively early phase of the disease, and finally showed red depigmented fundi in both eyes, as late sequelae of Vogt‐Koyanagi‐Harada disease. Routine ophthalmological examinations are required to check the recurrence of Vogt‐Koyanagi‐Harada disease in this patient as far as she would continue to use nivolumab.

In conclusions, Vogt‐Koyanagi‐Harada disease‐like posterior uveitis would develop in the process of immunological deregulation by nivolumab, anti‐PD‐1 antibody, in the treatment of metastatic malignant melanoma. Preceding administration of nivolumab might lead to an earlier onset of posterior uveitis as an adverse event of vemurafenib, BRAF inhibitor, in the flow of these two drugs for metastatic malignant melanoma. The development of Vogt‐Koyanagi‐Harada disease‐like posterior uveitis would be a clinical sign to suggest the efficacy of nivolumab in the treatment of metastatic malignant melanoma. Case reports of uveitis, as adverse events associated with immune checkpoint blockade, including CTLA‐4 inhibition (ipilimumab) and PD‐1 inhibition (nivolumab), have been rapidly emerging in the literature 13, 14, 15. Widespread use of ipilimumab and nivolumab in the near future would lead to finding clinical signs, such as Vogt‐Koyanagi‐Harada disease‐like posterior uveitis in this patient, to predict the drug efficacy at the clinical setting.

## Authorship

Both authors made substantial contributions to conception and design, analysis and interpretation of data, were involved in revising it critically for important intellectual content, and gave final approval of the version for submission. Both authors participated sufficiently in the work to take public responsibility for appropriate portions of the content.

## Consent

The patient has provided written consent for the case report to be published.

## Conflict of Interest

None declared.
